# An Insight into Ginsenoside Metabolite Compound K as a Potential Tool for Skin Disorder

**DOI:** 10.1155/2018/8075870

**Published:** 2018-06-25

**Authors:** En Hyung Kim, Wonnam Kim

**Affiliations:** ^1^Department of Dermatology, Cheil General Hospital and Women's Healthcare Center, Dankook University College of Medicine, Cheonan, Republic of Korea; ^2^Division of Pharmacology, College of Korean Medicine, Semyung University, Jecheon, Republic of Korea

## Abstract

Ginsenosides are the major bioactive natural compounds derived from* Panax ginseng*. Several studies report the pharmaceutical benefits of several ginsenosides, including antidementia, antitumor, and anti-inflammatory activity. Biotransformations by gut microbiome contribute to the biological function of these ginsenosides. After ingestion ginsenosides are hydrolyzed to Rg2, Rg3, compound K, and others by human gut flora. Compound K is considered the representative active metabolite after oral administration of ginseng or ginsenosides. Various studies report the diverse biological functions of compound K, such as antitumor, antidiabetic, antiallergic, and anti-inflammatory activity. Recent clinical trial and* in vitro* studies demonstrate the antiaging activities of ginsenosides in human skin. Ginsenosides have been considered as an important natural dermatological agent. In this review, we will cover the modern tools and techniques to understand biotransformation and delivery of compound K. Also the biological function of compound K on skin disorder and its potential dermatological application will be discussed.

## 1. Introduction

Ginseng, referring to the root and rhizome of* Panax ginseng*, is a representative medicinal herb commonly used thousands of years in Asia. Its active constituents are ginsenosides, a class of triterpenoid saponins, and are exclusively contained in* Panax* species and more than 150 ginsenosides are currently identified from ginseng roots, fruits, flower heads, leaves, and stems. [1]. Ginsenosides are divided into two main types by their chemical structures: protopanaxadiols (PPDs) and protopanaxatriols (PPTs) [2, 3]. PPD-type includes ginsenoside Rc, Rd, Rb1, and Rb2, while PPT-type includes ginsenosides Re, Rf, Rg1, and Rg2. There have been many reports describing the biological actions of several ginsenosides including antidementia, antitumor, and anti-inflammatory activities [4–6]. After ginseng or ginsenosides are orally consumed, compound K is considered the major functional component determined by plasma or organ [7]. The biotransformation by gut microbiome is closely linked to the diverse biological activities of these ginsenosides [8]. The deglycosylation of ginsenosides Rc, Rb1, and Rb2 by human gut bacteria produce compound K (20-*O*-*β*-(D-glucopyranosyl)-20(S)-protopanaxadiol) is an active metabolite. [9]. Numerous experimental studies of compound K have shown the antitumor, antidiabetic, antiallergic, and anti-inflammatory effects [10, 11].

For thousands of years, the benefits of ginseng are well known to treat a wide variety of diseases. It also has been used to improve the overall condition of skin [12]. Chinese traditional medicine textbooks describe its ability as a topical treatment for wounds, atopic dermatitis, and other inflammatory skin symptoms [13]. Recently, there have been a few studies to clarify the efficacy of ginseng in skin [12]. A number of human and animal studies have demonstrated that dermatological formulations comprising crude extracts of ginseng show positive benefits on the skin [14–16]. Other reports indicate that ginsenoside Rb1 [17] and Rb2 [15] stimulates the recovery of burn injury, and topical treatment of compound K may help to avoid or ways to improve skin deteriorations with age caused by loss of hyaluronan in human skin [18]. This review summarizes the current understanding of compound K, and its dermatological and cosmeceutical benefits.

## 2. Overview of Compound K

### 2.1. Role of Intestinal Microbiota

The human intestine is populated with a large community of microorganisms and is a site where they affect human health as well as drugs' fate [19]. The intestinal microbiota, represented as a “microbial organ,” can contribute important roles in the metabolic function of drugs and affect the stability and oral bioavailability of drugs [20–22]. Gut is an emerging therapeutic target, especially for herbal products and dietary supplements [21, 23]. As herbal products are mostly consumed orally, it can inevitably affect the gut microbiota in different ways [24].

First, herbs may change the population of the gut microbiota to maintain a homeostatic balance [25]. Green tea has been reported to exert anti-inflammatory and antiobesity effects and also have been found to change composition of the gut microbiota [26, 27]. Seo's group reported that fermented green tea extract restored the ratio changes in* Firmicutes/Bacteroidetes* and* Bacteroides/Prevotella* induced by high-fat diet, which may explain the underlying mechanism that improves obesity and its related disorders [28]. Axling's group added one strain of* Lactobacillus plantarum* with green tea powder and found reduction in inflammatory markers affected from high-fat diet and expansion in gut microbial diversity which may act as a positive health factor [29]. Several studies have reported the potent anticancer activities of* Gynostemma pentaphyllum* (Gp) [30]. Chen's group first demonstrated that Gp saponins (GpS) elicit anticancer responses on tumor xenograft models [31]. They also showed that tumor implants significantly altered the gut microbiota compositions assessed with ERIC-PCR and 16S pyrosequencing procedures [31]. Interestingly, GpS treatment augmented the relative abundance of probiotics such as* Clostridium cocleatum* and* Bacteroides acidifaciens *modulated by tumor implantation [31].

Second, herbs may undergo gut microbiota-mediated bioconversion process influencing the drug metabolism [24].* Coptis chinensis* contains alkaloids such as berberine, which has been widely studied due to its potent antimicrobial, antioxidant, anti-inflammatory, anticancer, antidiabetic, neuroprotective, nephroprotective, and hepatoprotective activity [32]. However, berberine exhibits poor water solubility partly contributing to its low bioavailability and poor intestinal absorption [33]. A recent study by Feng's group suggests that interaction between the gut microbiota and berberine enhances its absorption [34]. In fact, the gut microbiota transforms berberine to dihydroberberine, a 5-fold higher absorbable form, and if treated with antibiotics the level of gut flora was lowered and as a result the plasma concentrations of berberine were lowered, reducing its therapeutic efficacy [34]. Ginsenosides from* Panax ginseng* are involved in modulating numerous physiological functions [35]. Ginsenoside Rb1, a 20(S)-protopanaxadiol (PPD) type ginsenoside, one of the important components in ginseng total saponins, possesses various beneficial effects [36]. However, biotransformation may be required for ginsenoside Rb1 due to its poor membrane permeability and higher susceptibility to degradation [35]. Increased biological effects of ginsenoside Rb1 is mediated by metabolites metabolized by human intestinal microbes [9].

### 2.2. Biotransformation to Compound K

**Figure 1:**
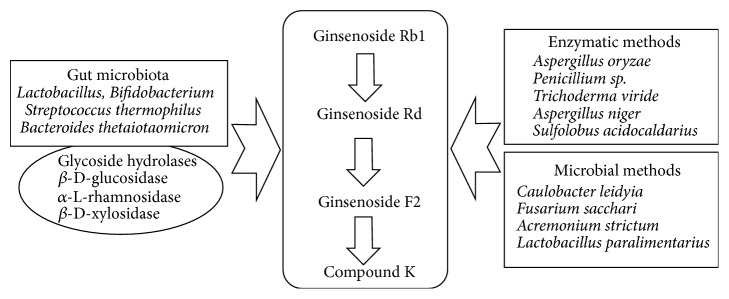
Biotransformation to compound K.

After ginsenosides are consumed orally, ginsenosides are metabolized by deglycosylation reactions [37, 38]. Gut microbiota including* Lactobacillus*,* Bifidobacterium*,* Streptococcus thermophilus*, and* Bacteroides thetaiotaomicron* possess different types of glycosidases, such as *β*-D-glucosidase, *α*-L-rhamnosidase, and *β*-D-xylosidase [37]. Ginsenoside Rb1 undergoes stepwise hydrolysis of the sugar moieties to secondary ginsenosides or aglycone by *β*-D-glucosidase [37]. Ginsenoside Rb1 is rapidly hydrolyzed to ginsenoside Rd and then in a rate-limiting step deglycosylated to ginsenoside F2 and further converts to the compound K through hydrolysis [39] (Figure 1). Due to its diverse biological activities, compound K has attracted growing interests in methods on how to increase its quantity. Conventional chemical approaches, such as heating, hydrolysis with weak acid, and cleavage by alkali, have been studied; however, microbial or enzymatic conversion methods are considered more favorable due to their prominent selectivity, moderate reaction conditions, and environmental compatibility [40–45]. Enzymatic methods to produce compound K use lactase, cellulose, and *β*-D-glycosidase, which are purified from* Aspergillus oryzae*,* Penicillium sp*.,* Trichoderma viride*,* Aspergillus niger*, and* Sulfolobus acidocaldarius* [46–48] (Figure 1). Microbial methods using crude enzymes from* Caulobacter leidyia*,* Fusarium sacchari*,* Acremonium strictum*, and* Lactobacillus paralimentarius* were reported to achieve compound K [49–52] (Figure 1).

To understand the pharmacokinetics of compound K,* in vitro* and* in vivo* studies have been processed by dose-dependent oral administration [53]. An open trial study on single oral dose of red ginseng product shows that absorption of compound K is not affected by its parent compound, ginsenoside Rb1, except the fact that the delay to reach the maximum serum concentration explains the required transformation process [54]. Moreover, a human pharmacokinetic study comparing the pharmacokinetic parameters of compound K between fermented and nonfermented red ginseng indicates that fermented group absorbed higher and faster in greater amounts than nonfermented group [55]. Recent human pharmacokinetic data from single and multiple dose studies of compound K suggest the influence of sex and food related factors [56, 57].

### 2.3. Advances in Delivery of Compound K

The therapeutic use of compound K may be restricted because of poor aqueous solubility, low membrane permeability, and P-glycoprotein mediated efflux [58]. To improve the solubility and stability of active constituents several approaches were developed, including polymeric nanoparticles, solid lipid nanoparticles, liquid crystal systems, precursors systems for liquid crystals, liposomes, and microemulsions [59]. Polyethylene glycol (PEG) is a widely used hydrophilic, nonionic, and nontoxic polymeric carrier in drug delivery systems [60]. Surface modification using PEG increases water solubility protects from proteolytic degradation, prolongs circulation half-life in blood, reduces systemic toxicity, and improved therapeutic indices [61]. Mathiyalagan's group generated a pH-sensitive PEG-compound K conjugate through an acid-labile ester-linkage that enhanced water solubility of compound K [62]. They also covalently conjugated hydrophobic compound K with hydrophilic glycol chitosan backbone by an acid-labile linkage to improve aqueous solubility and targeted delivery [63]. The nanoparticles were stable under physiological pH, whereas they degraded easily under acidic pH that mimics the intracellular pH levels [63].

D-*α*-Tocopheryl polyethylene glycol 1000 succinate monoester (vitamin E TPGS or simply TPGS) possesses the benefits of both promoting solubility and suppressing P-glycoprotein [64]. TPGS based formulation could increase solubility, permeability, and stability, prolong the half-life, and improve the cellular uptake of the drug [65, 66]. Yang's group prepared ginsenoside compound K-loaded TPGS-modified liposomes (GCKT-liposomes) to increase the solubility and targeting capability of compound K [67]. The GCKT-liposomes significantly increased the cellular uptake and its cytotoxicity* in vitro* and also showed higher antitumor efficacy by grafting A549 cells into nude mice* in vivo* [67]. Zhang's group used a novel ascorbyl palmitate (AP)/TPGS mixed micellar system with compound K and reported an increased antitumor effect* in vitro* [68]. The compound K-loaded AP/TPGS mixed micelles significantly enhanced cellular uptake and tumor targeting resulting in decreased tumor volumes in the A549 xenograft models [68]. Furthermore, Yang's group used TPGS/PEG-poly(*ε*-caprolactone) (PCL) mixed micelles with compound K to increase the water solubility and the cellular uptake in tumor tissue [69]. This carrier system enhanced the antitumor effect of compound K by promoting apoptosis and inhibiting cell invasion and migration in A549 and PC-9 cells [69].

## 3. Biological Activity of Skin

### 3.1. Dermatological Activity

Pruritus or itching is an unpleasant skin sensation that frequently provokes scratching and is generally relevant with primary skin lesions such as urticaria, atopic dermatitis, or systemic diseases such as cholestasis and uraemia [70]. A number of chemical agents, like proteases, cytokines, prostaglandins, histamine, neuropeptide substance P, and bile salts, can act as pruritogens [71]. Shin's group investigated the antipruritic effects of ginsenoside Rb1 and compound K in response to compound 48/80, substance P, and histamine using behavioral mouse model for itch [70]. Compound K treatment reduced scratching behaviors and skin vascular permeability activated by compound 48/80, substance P, and histamine [70].

The anticancer effects of compound K have been investigated by focusing on skin related cancer. Lee's group studied the effects of compound K on tumor progression and mediated molecular changes [72]. Tumor progression is regulated by elevation of ornithine decarboxylase (ODC), free radicals, reactive oxygen species (ROS), COX-2, and NF-*κ*B activity [73, 74].

To induce mouse ear edema, prototype tumor promoter 12-*O*-tetradecanoylphorbol-13-acetate (TPA) was applied [72]. Compound K pretreatment inhibited the TPA induced activity of COX-2 and ODC by interfering with extracellular signal regulated kinase (ERK) and nuclear factor-*κ*B (NF-kB) pathway [72]. Melanoma is notoriously resistant to most approaches to treat the aggressive and lethal skin cancer. A series of functional, biochemical, and gene sequencing indicated that melanoma cells frequently acquire chemoresistance by exploiting their intrinsic apoptosis resistance and by reprogramming pathways associated with cell proliferation and survival during melanoma progression [75]. Development of highly potent and specific compounds is urgently needed to block signaling networks critical for melanoma [76]. Kang's group reported the mechanism of action responsible for the antitumor effect of compound K in melanoma progression [77]. Compound K appears to inhibit melanoma cell proliferation and growth in anchorage independent conditions [77]. Also, compound K treatment activated AMPK/JNK signaling and induced cell death mediated by autophagy and apoptosis [77].

Studies have reported the effect of compound K on inflammatory skin conditions. Atopic dermatitis (AD), or atopic eczema, is a common chronic, relapsing, and often intensely pruritic inflammatory disorder of the skin [78]. Kim's group demonstrated that compound K treatment in NC/Nga mice attenuates* Dermatophagoides farinae* body extract (DFE) antigen-induced AD-like symptoms, including increased dermatitis severity score, ear thickness, and infiltration of inflammatory cells in the skin lesions [79]. These effects were regulated by decrease in serum levels of macrophage derived chemokine and production of T cell-derived proinflammatory cytokines in cultured* ex vivo* splenocytes, including IFN-*γ*, GM-CSF, TNF-*α*, IL-4, IL-5, IL-10, and IL-12 [79].

As described above, compound K is a promising therapeutic approach for inflammatory related skin disorders; however there are limited experimental studies and clinical trials to fully understand and evaluate the pharmacological activities.

### 3.2. Cosmeceutical Activity

The nutritional benefits of ginseng on skin health are characterized by activating skin metabolism due to enhanced blood flow and cell proliferation which may be related to the antiaging capabilities [80]. Many studies support that ginsenosides elicit antiaging effects by free radical scavenging and suppressing lipid peroxidation [81].

Hyaluronic acid (HA) also called hyaluronan is an, evolutionarily conserved, abundant linear polysaccharides [82]. Since its discovery in 1934, HA has been widely applicable in the field of cutaneous wound repair, neurosurgery, and cosmetic practice [83]. The HA synthesis and turnover have been shown to decline with age [84]. This decline is important for decreased turgidity, wrinkling, reduced elasticity, and weakened support for microvessels in aged skin [85]. HA is synthesized by three different plasma membrane bound hyaluronan synthase (HAS) enzymes, namely, HAS1, HAS2, and HAS3 [86]. Kim's group treated immortalized keratinocyte, HaCaT cells, with compound K, and examined the gene expressions of 100 transcripts using cDNA microarray technology [18]. HAS2 gene expression was upregulated significantly by compound K and enhanced HA content in aged skin by HA synthesis [18]. A later study by Lim's group reported the underlying mechanism for augmented HA production by compound K [87]. The study provides evidence that the production of HA induced by compound K is mediated by Src-dependent Akt and ERK activation, but not EGFR or Ca2+ changes [87].

Exposure to ultraviolet (UV) radiation on human skin is highly correlated with skin diseases [88]. Prolonged UV exposure affects many different biological alterations that are directly or indirectly associated with skin aging and cancer incidents [89]. UVA comprises most of the UV radiation that reaches the earth's surface; chronic exposure to UVA penetrates deeply through into the human skin and damages the underlying support by the dermis causing premature photoaging and forms wrinkles and sagging skin [88, 90, 91]. UV radiation activates particular matrix metalloproteinase (MMP) family members that mediate collagen degradation that is observed in photoaged skin [92]. Dermal fibroblasts express matrix metalloproteinase-1 (MMP-1) by exposure to both UVA and B [93]. He's group treated UVA-irradiated fibroblasts with compound K and showed that type I collagen production increased while, under the same experimental conditions, MMP-1 activity decreased [94]. UVB irradiation stimulates MMP expression by regulating transcription factors, such as activator protein-1 (AP-1) and NF-*κ*B in the epidermis [95]. The mitogen-activated protein kinase (MAPK) signaling pathway results in expression of AP-1 activation; depending on the cell type I*κ*B kinase (IKK), phosphoinositide 3 kinase- (PI3K-) Akt and p38 MAPK have been associated with NF-*κ*B activation [96, 97]. Thus, investigation of compounds targeting UVB-induced MMP levels and/or its upstream regulators may offer advantages to prevent and treat skin aging [98]. Shin's group reported the inhibitory effect of compound K on MMP-1 levels in human dermal fibroblasts (HDFs) by UV, which is due to the effect of adenosine monophosphate-activated protein kinase (AMPK) as a downstream of the cAMP-dependent protein kinase- (PKA-) liver kinase B1 (LKB1) pathway [99]. Damaged DNA by UVB causes cyclobutane pyrimidine dimers (CPDs), while UVA exposure mostly damages indirectly through ROS generation [93]. Most of UVB-induced DNA damage in humans is removed by the response of nucleotide excision repair (NER) pathway [100]. Cai's group reported that compound K augment UVB induced cell death in HaCaT cells [101]. Compound K, by DNA repair induction, caused a notable reduction against CPD in later stages after UVB irradiation [101]. Compound K augmented the decrease in specific components of the NER complex, such as XPC and ERCC1 by UVB [101]. Hong's group used BIOGF1K, a fraction rich in compound K, to study the antiphotoaging effect induced by UVB irradiation on NIH3T3 and B16F10 cells [102]. BIOGF1K inhibited the UVB-induced apoptosis, morphological changes, and melanin secretion [102]. Skin inflammation is closely linked to skin aging because inflammation induced inflammatory cytokines and halogenated tyrosine increases protein denaturation resulting in skin aging [103]. Lee's group showed that compound K inhibits TNF-*α* induced MMP-1 secretion, a characteristic feature of skin aging in human [104]. The ability of compound K to inhibit the degradation of collagen in human fibroblasts by TNF-*α* stimulated MMP-1 secretion is regulated by inactivation of c-Src/EGFR-dependent ERK/AP-1 signal pathways [104].

As a cosmeceutical, skin (percutaneous, dermal) absorption of compound K is an important factor when applied topically. However, hydrophilic properties of glycosides due to the glycosyl group limit skin permeability which is disadvantageous for cosmetics purposes [105]. The aglycones are more hydrophobic and can effectively permeate the skin [106]. Therefore, enhancing biological activity of extracts by glycosides hydrolyzed into aglycones has attracted much attention [105]. Previous study has mentioned the antiallergic effects of compound K through mast cell via a membrane stabilizing activity [107]. Thus skin problems such as irritation and sensitization may be lower in compound K. A significant amount of research has been conducted to evaluate the pharmacological effects of compound K, to expand the scope of its potential applications further clinical studies will be required.

*Summary*. Medicinal use and safety of ginseng have been recognized for thousands of years with evidence suggesting the antiaging activities, such as wrinkle reduction and sun protection of ginseng extract and ginsenosides. To express the pharmacological actions of ginsenosides, orally administered ginseng is biotransformed by intestinal microbiota into compound K. However as a dermatological agent, compound K is primarily used topically and due to the omission of intestinal absorption and biotransformation, strategies to enhance skin absorption are an important step. Moreover, the dermatological effect of compound K at the molecular level is poorly understood. Therefore, a better mechanistic understanding of compound K can lead to more effective delivery method. Also the safety of compound K when applied frequently onto skin still remains unclear. Further study to improve skin penetration and clinical tests for efficacy and safety of compound K is needed for its commercial use.

## References

[1] Christensen L. P. (2008). Chapter 1 ginsenosides: chemistry, biosynthesis, analysis, and potential health effects. *Advances in Food and Nutrition Research*.

[2] Chu S.-F., Zhang J.-T. (2009). New achievements in ginseng research and its future prospects. *Chinese Journal of Integrative Medicine*.

[3] Smith I., Williamson E. M., Putnam S., Farrimond J., Whalley B. J. (2014). Effects and mechanisms of ginseng and ginsenosides on cognition. *Nutrition Reviews*.

[4] Huang Q., Wang T., Wang H.-Y. (2017). Ginsenoside Rb2 enhances the anti-inflammatory effect of *ω*-3 fatty acid in LPS-stimulated RAW264.7 macrophages by upregulating GPR120 expression. *Acta Pharmacologica Sinica*.

[5] Wang P., Du X., Xiong M. (2016). Ginsenoside Rd attenuates breast cancer metastasis implicating derepressing microRNA-18a-regulated Smad2 expression. *Scientific Reports*.

[6] Li F., Wu X., Li J., Niu Q. (2016). Ginsenoside Rg1 ameliorates hippocampal long-term potentiation and memory in an Alzheimer's disease model. *Molecular Medicine Reports*.

[7] Hasegawa H. (2004). Proof of the mysterious efficacy of ginseng: basic and clinical trials: metabolic activation of ginsenoside: deglycosylation by intestinal bacteria and esterification with fatty acid. *Journal of Pharmacological Sciences*.

[8] Wakabayashi C., Murakami K., Hasegawa H., Murata J., Saiki I. (1998). An intestinal bacterial metabolite of ginseng protopanaxadiol saponins has the ability to induce apoptosis in tumor cells. *Biochemical and Biophysical Research Communications*.

[9] Wakabayashi C., Hasegawa H., Murata J., Saiki I. (1997). In vivo antimetastatic action of ginseng protopanaxadiol saponins is based on their intestinal bacterial metabolites after oral administration. *Oncology Research : Featuring Preclinical and Clinical Cancer Therapeutics*.

[10] Jia L., Zhao Y., Liang X. (2009). Current evaluation of the millennium phytomedicine—Ginseng (II): collected chemical entities, modern pharmacology, and clinical applications emanated from traditional chinese medicine. *Current Medicinal Chemistry*.

[11] Radad K., Moldzio R., Rausch W.-D. (2011). Ginsenosides and their CNS Targets. *CNS Neuroscience & Therapeutics*.

[12] Kim K. (2015). Effect of ginseng and ginsenosides on melanogenesis and their mechanism of action. *Journal of Ginseng Research*.

[13] Kimura Y., Sumiyoshi M., Sakanaka M. (2012). Effects of ginsenoside R b 1 on skin changes. *Journal of Biomedicine and Biotechnology*.

[14] Keum Y.-S., Park K.-K., Lee J.-M. (2000). Antioxidant and anti-tumor promoting activities of the methanol extract of heat-processed ginseng. *Cancer Letters*.

[15] Choi S. (2002). Epidermis proliferative effect of the Panax ginseng ginsenoside Rb 2. *Archives of Pharmacal Research*.

[16] Chang L. K., Whitaker D. C. (2001). The impact of herbal medicines on dermatologic surgery. *Dermatologic Surgery*.

[17] Kimura Y., Sumiyoshi M., Kawahira K., Sakanaka M. (2006). Effects of ginseng saponins isolated from Red Ginseng roots on burn wound healing in mice. *British Journal of Pharmacology*.

[18] Kim S., Kang B. Y., Cho S. Y. (2004). Compound K induces expression of hyaluronan synthase 2 gene in transformed human keratinocytes and increases hyaluronan in hairless mouse skin. *Biochemical and Biophysical Research Communications*.

[19] Nicholson J. K., Holmes E., Wilson I. D. (2005). Gut microorganisms, mammalian metabolism and personalized health care. *Nature Reviews Microbiology*.

[20] Lederberg J. (2000). The dawning of molecular genetics. *Trends in Microbiology*.

[21] Jia W., Li H., Zhao L., Nicholson J. K. (2008). Gut microbiota: A potential new territory for drug targeting. *Nature Reviews Drug Discovery*.

[22] Kang M. J., Kim H. G., Kim J. S. (2013). The effect of gut microbiota on drug metabolism. *Expert Opinion on Drug Metabolism & Toxicology*.

[23] Li H., Zhou M., Zhao A., Jia W. (2009). Traditional Chinese medicine: balancing the gut ecosystem. *Phytotherapy Research*.

[24] Chen F., Wen Q., Jiang J. (2016). Could the gut microbiota reconcile the oral bioavailability conundrum of traditional herbs?. *Journal of Ethnopharmacology*.

[25] Zhao L., Nicholson J. K., Lu A. (2012). Targeting the human genome-microbiome axis for drug discovery: Inspirations from global systems biology and traditional Chinese medicine. *Journal of Proteome Research*.

[26] Cunha C. A., Lira F. S., Rosa Neto J. C. (2013). Green tea extract supplementation induces the lipolytic pathway, attenuates obesity, and reduces low-grade inflammation in mice fed a high-fat diet. *Mediators of Inflammation*.

[27] Jin J.-S., Touyama M., Hisada T., Benno Y. (2012). Effects of green tea consumption on human fecal microbiota with special reference to Bifidobacterium species. *Microbiology and Immunology*.

[28] Seo D.-B., Jeong H. W., Cho D. (2015). Fermented green tea extract alleviates obesity and related complications and alters gut microbiota composition in diet-induced obese mice. *Journal of Medicinal Food*.

[29] Axling U., Olsson C., Xu J. (2012). Green tea powder and Lactobacillus plantarum affect gut microbiota, lipid metabolism and inflammation in high-fat fed C57BL/6J mice. *Journal of Nutrition and Metabolism*.

[30] Li Y., Lin W., Huang J., Xie Y., Ma W. (2016). Anti-cancer effects of Gynostemma pentaphyllum (Thunb.) Makino (Jiaogulan). *Chinese Medicine*.

[31] Chen L., Tai W. C. S., Brar M. S., Leung F. C. C., Hsiao W. L. W. (2015). Tumor grafting induces changes of gut microbiota in athymic nude mice in the presence and absence of medicinal Gynostemma saponins. *PLoS ONE*.

[32] Kumar A., Ekavali, Chopra K., Mukherjee M., Pottabathini R., Dhull D. K. (2015). Current knowledge and pharmacological profile of berberine: An update. *European Journal of Pharmacology*.

[33] Liu Y., Zhang L., Song H., Ji G. (2013). Update on berberine in nonalcoholic Fatty liver disease. *Evidence-Based Complementary and Alternative Medicine*.

[34] Feng R., Shou J., Zhao Z. (2015). Transforming berberine into its intestine-absorbable form by the gut microbiota. *Scientific Reports*.

[35] Leung K., Wong A. (2010). Pharmacology of ginsenosides: a literature review. *Chinese Medicine*.

[36] Ahmed T., Raza S. H., Maryam A. (2016). Ginsenoside Rb1 as a neuroprotective agent: a review. *Brain Research Bulletin*.

[37] An K., Shengjie Z., Jinjun S., Liuqing D. Gut microbiota-mediated deglycosylation of ginsenoside Rb1 in rats: in vitro and in vivo insights from quantitative ultra-performance liquid chromatography-mass spectrometry analysis. *Analytical Methods*.

[38] Zhang J., Zhou F., Lu M. (2012). Pharmacokinetics-pharmacology disconnection of herbal medicines and its potential solutions with cellular pharmacokinetic-pharmacodynamic strategy. *Current Drug Metabolism*.

[39] Niu T., Smith D. L., Yang Z. (2013). Bioactivity and bioavailability of ginsenosides are dependent on the glycosidase activities of the A/J mouse intestinal microbiome defined by pyrosequencing. *Pharmaceutical Research*.

[40] Kim W. Y., Kim J. M., Han S. B. (2000). Steaming of ginseng at high temperature enhances biological activity. *Journal of Natural Products*.

[41] Han B. H., Park M. H., Han Y. N. (1982). Degradation of ginseng saponins under mild acidic conditions. *Planta Medica*.

[42] Chen Y., Nose M., Ogihara Y. (1987). Alkaline cleavage of ginsenosides. *Chemical & Pharmaceutical Bulletin*.

[43] Bae E., Park S., Kim D. (2000). Constitutive .BETA.-Glucosidases Hydrolyzing Ginsenoside Rb1 and Rb2 from Human Intestinal Bacteria. *Biological & Pharmaceutical Bulletin*.

[44] Yan Q., Zhou X.-W., Zhou W., Li X.-W., Feng M.-Q., Zhou P. (2008). Purification and properties of a novel *β*-glucosidase, hydrolyzing ginsenoside Rb1 to CK, from Paecilomyces Bainier. *Journal of Microbiology and Biotechnology*.

[45] Yang X.-D., Yang Y.-Y., Ouyang D.-S., Yang G.-P. (2015). A review of biotransformation and pharmacology of ginsenoside compound K. *Fitoterapia*.

[46] Hu J.-N., Zhu X.-M., Lee K.-T. (2008). Optimization of ginsenosides hydrolyzing *β*-glucosidase production from Aspergillus niger using response surface methodology. *Biological & Pharmaceutical Bulletin*.

[47] Ko S.-R., Suzuki Y., Suzuki K., Choi K.-J., Cho B.-G. (2007). Marked production of ginsenosides Rd, F2, Rg3, and compound K by enzymatic method. *Chemical & Pharmaceutical Bulletin*.

[48] Noh K.-H., Oh D.-K. (2009). Production of the rare ginsenosides compound K, compound Y, and compound Mc by a thermostable *β*-glycosidase from Sulfolobus acidocaldarius. *Biological & Pharmaceutical Bulletin*.

[49] Cheng L.-Q., Kim M. K., Lee J.-W., Lee Y.-J., Yang D.-C. (2006). Conversion of major ginsenoside Rb1 to ginsenoside F2 by Caulobacter leidyia. *Biotechnology Letters*.

[50] Han Y., Sun B., Hu X. (2007). Transformation of bioactive compounds by Fusarium sacchari fungus isolated from the soil-cultivated ginseng. *Journal of Agricultural and Food Chemistry*.

[51] Chen G.-T., Yang M., Song Y. (2008). Microbial transformation of ginsenoside Rb1 by Acremonium strictum. *Applied Microbiology and Biotechnology*.

[52] Quan L.-H., Kim Y.-J., Li G. H., Choi K.-T., Yang D.-C. (2013). Microbial transformation of ginsenoside Rb1 to compound K by Lactobacillus paralimentarius. *World Journal of Microbiology and Biotechnology*.

[53] Paek I. B., Moon Y., Kim J. (2006). Pharmacokinetics of a ginseng saponin metabolite compound K in rats. *Biopharmaceutics & Drug Disposition*.

[54] Kim H.-K. (2013). Pharmacokinetics of ginsenoside Rb1 and its metabolite compound K after oral administration of Korean Red Ginseng extract. *Journal of Ginseng Research*.

[55] Choi I.-D., Ryu J.-H., Lee D.-E. (2016). Enhanced Absorption Study of Ginsenoside Compound K (20- O - *β* -(D-Glucopyranosyl)-20(S)-protopanaxadiol) after Oral Administration of Fermented Red Ginseng Extract (HYFRG ™) in Healthy Korean Volunteers and Rats. *Evidence-Based Complementary and Alternative Medicine*.

[56] Chen L., Zhou L., Huang J. (2018). Single- and Multiple-Dose Trials to Determine the Pharmacokinetics, Safety, Tolerability, and Sex Effect of Oral Ginsenoside Compound K in Healthy Chinese Volunteers. *Frontiers in Pharmacology*.

[57] Chen L., Zhou L., Wang Y. (2017). Food and sex-related impacts on the pharmacokinetics of a single-dose of ginsenoside compound K in healthy subjects. *Frontiers in Pharmacology*.

[58] Yang Z., Wang J.-R., Niu T. (2012). Inhibition of P-glycoprotein leads to improved oral bioavailability of compound K, an anticancer metabolite of red ginseng extract produced by gut microflora. *Drug Metabolism and Disposition*.

[59] Bonifácio B. V., da Silva P. B., Ramos M. A. D. S., Negri K. M. N., Bauab T. M., Chorilli M. (2014). Nanotechnology-based drug delivery systems and herbal medicines: a review. *International Journal of Nanomedicine*.

[60] Knop K., Hoogenboom R., Fischer D., Schubert U. S. (2010). Poly(ethylene glycol) in drug delivery: pros and cons as well as potential alternatives. *Angewandte Chemie International Edition*.

[61] Mishra P., Nayak B., Dey R. K. (2016). PEGylation in anti-cancer therapy: An overview. *Asian Journal of Pharmaceutical Sciences*.

[62] Mathiyalagan R., Subramaniyam S., Kim Y. J. (2014). Synthesis and pharmacokinetic characterization of a pH-sensitive polyethylene glycol ginsenoside CK (PEG-CK) conjugate. *Bioscience, Biotechnology, and Biochemistry*.

[63] Mathiyalagan R., Subramaniyam S., Kim Y. J., Kim Y.-C., Yang D. C. (2014). Ginsenoside compound K-bearing glycol chitosan conjugates: Synthesis, physicochemical characterization, and in vitro biological studies. *Carbohydrate Polymers*.

[64] Duhem N., Danhier F., Préat V. (2014). Vitamin E-based nanomedicines for anti-cancer drug delivery. *Journal of Controlled Release*.

[65] Collnot E.-M., Baldes C., Wempe M. F. (2006). Influence of vitamin E TPGS poly(ethylene glycol) chain length on apical efflux transporters in Caco-2 cell monolayers. *Journal of Controlled Release*.

[66] Prashant C., Dipak M., Yang C.-T., Chuang K.-H., Jun D., Feng S.-S. (2010). Superparamagnetic iron oxide - Loaded poly (lactic acid)-d-*α*-tocopherol polyethylene glycol 1000 succinate copolymer nanoparticles as MRI contrast agent. *Biomaterials*.

[67] Yang L., Xin J., Zhang Z. (2016). TPGS-modified liposomes for the delivery of ginsenoside compound K against non-small cell lung cancer: formulation design and its evaluation in vitro and in vivo. *Journal of Pharmacy and Pharmacology*.

[68] Zhang Y., Tong D., Che D. (2017). Ascorbyl palmitate/D-*α*-tocopheryl polyethylene glycol 1000 succinate monoester mixed micelles for prolonged circulation and targeted delivery of compound K for antilung cancer therapy in vitro and in vivo. *International Journal of Nanomedicine*.

[69] Yang L., Zhang Z., Hou J. (2017). Targeted delivery of ginsenoside compound K using TPGS/PEG-PCL mixed micelles for effective treatment of lung cancer. *International Journal of Nanomedicine*.

[70] Shin Y.-W., Kim D.-H. (2005). Antipruritic effect of ginsenoside Rb1 and compound K in scratching behavior mouse models. *Journal of Pharmacological Sciences*.

[71] Yonova D. (2007). Pruritus in certain internal diseases. *Hippokratia*.

[72] Lee J.-Y., Shin J.-W., Chun K.-S. (2005). Antitumor promotional effects of a novel intestinal bacterial metabolite (IH-901) derived from the protopanaxadiol-type ginsenosides in mouse skin. *Carcinogenesis*.

[73] Fujiki H., Suganuma M., Komori A. (1994). A new tumor promotion pathway and its inhibitors. *Cancer Detect Prev*.

[74] Dolcet X., Llobet D., Pallares J., Matias-Guiu X. (2005). NF-kB in development and progression of human cancer. *Virchows Archiv*.

[75] Soengas M. S., Lowe S. W. (2003). Apoptosis and melanoma chemoresistance. *Oncogene*.

[76] Rozenblat S., Grossman S., Bergman M., Gottlieb H., Cohen Y., Dovrat S. (2008). Induction of G2/M arrest and apoptosis by sesquiterpene lactones in human melanoma cell lines. *Biochemical Pharmacology*.

[77] Kang S., Kim J.-E., Song N. R. (2014). The ginsenoside 20-O-*β*-D-glucopyranosyl-20(S)-protopanaxadiol induces autophagy and apoptosis in human melanoma via AMPK/JNK phosphorylation. *PLoS ONE*.

[78] Tollefson M. M., Bruckner A. L., Cohen B. A. (2014). Atopic dermatitis: skin-directed management. *Pediatrics*.

[79] Kim J. R., Choi J., Kim J. (2014). 20-O-*β*-d-glucopyranosyl-20(S)-protopanaxadiol-fortified ginseng extract attenuates the development of atopic dermatitis-like symptoms in NC/Nga mice. *Journal of Ethnopharmacology*.

[80] Lee O.-S., Kang H.-H., Han S.-H. Oriental herbs in cosmetics: Plant extracts are reviewed for their potential as cosmetic ingredients. *Cosmetics and toiletries*.

[81] Aburjai T., Natsheh F. M. (2003). Plants used in cosmetics. *Phytotherapy Research*.

[82] Robert L. (2015). Hyaluronan, a truly ‘youthful’ polysaccharide. Its medical applications. *Pathologie Biologie*.

[83] Price R. D., Berry M. G., Navsaria H. A. (2007). Hyaluronic acid: the scientific and clinical evidence. *Journal of Plastic, Reconstructive & Aesthetic Surgery*.

[84] Robert L., Robert A.-M., Renard G. (2010). Biological effects of hyaluronan in connective tissues, eye, skin, venous wall. Role in aging. *Pathologie Biologie*.

[85] Baumann L. (2007). Skin ageing and its treatment. *The Journal of Pathology*.

[86] Itano N., Kimata K. (2002). Mammalian hyaluronan synthases. *IUBMB Life*.

[87] Lim T.-G., Jeon A. J., Yoon J. H. (2015). 20-O-*β*-D-glucopyranosyl-20(S)-protopanaxadiol, a metabolite of ginsenoside Rb1, enhances the production of hyaluronic acid through the activation of ERK and Akt mediated by Src tyrosin kinase in human keratinocytes. *International Journal of Molecular Medicine*.

[88] Svobodova A., Psotova J., Walterova D. (2003). Natural phenolics in the prevention of UV-induced skin damage: a review. *Biomed Pap Med Fac Univ Palacky Olomouc Czech Repub*.

[89] Bernstein E. F., Yue Qiu Chen, Kopp J. B. (1996). Long-term sun exposure alters the collagen of the papillary dermis: comparison of sun-protected and photoaged skin by Northern analysis immunohistochemical staining, and confocal laser scanning microscopy. *Journal of the American Academy of Dermatology*.

[90] Krutmann J. (2001). The role of UVA rays in skin aging. *European Journal of Dermatology*.

[91] Ichihashi M., Ando H., Yoshida M., Niki Y., Matsui M. (2009). Photoaging of the skin. *The Journal of Anti-Aging Medicine*.

[92] Quan T., Qin Z., Xia W., Shao Y., Voorhees J. J., Fisher G. J. (2009). Matrix-degrading metalloproteinases in photoaging. *The Journal of Investigative Dermatology, Symposium Proceedings*.

[93] Dong K. K., Damaghi N., Picart S. D. (2008). UV-induced DNA damage initiates release of MMP-1 in human skin. *Experimental Dermatology*.

[94] He D., Sun J., Zhu X., Nian S., Liu J. (2011). Compound K increases type I procollagen level and decreases matrix metalloproteinase-1 activity and level in ultraviolet-A-irradiated fibroblasts. *Journal of the Formosan Medical Association*.

[95] Cooper S. J., Bowden G. T. (2007). Ultraviolet B regulation of transcription factor families: roles of nuclear factor-kappa B (NF-kappaB) and activator protein-1 (AP-1) in UVB-induced skin carcinogenesis. *Current Cancer Drug Targets*.

[96] Karin M. (1995). The regulation of AP-1 activity by mitogen-activated protein kinases. *The Journal of Biological Chemistry*.

[97] Madrid L. V., Mayo M. W., Reuther J. Y., Baldwin A. S. (2001). Akt stimulates the transactivation potential of the RelA/p65 Subunit of NF-*κ* B through utilization of the I*κ* B kinase and activation of the mitogen-activated protein kinase p38. *The Journal of Biological Chemistry*.

[98] Hwang B.-M., Noh E.-M., Kim J.-S. (2013). Decursin inhibits UVB-induced MMP expression in human dermal fibroblasts via regulation of nuclear factor-*κ*B. *International Journal of Molecular Medicine*.

[99] Shin D. J., Kim J.-E., Lim T.-G. (2014). 20-O-*β*-d-glucopyranosyl-20(S)-protopanaxadiol suppresses UV-induced MMP-1 expression through AMPK-mediated mTOR inhibition as a downstream of the PKA-LKB1 pathway. *Journal of Cellular Biochemistry*.

[100] Fleck O., Nielsen O. (2004). DNA repair. *Journal of Cell Science*.

[101] Cai B.-X., Luo D., Lin X.-F., Gao J. (2008). Compound K suppresses ultraviolet radiation-induced apoptosis by inducing DNA repair in human keratinocytes. *Archives of Pharmacal Research*.

[102] Hong Y. H., Kim D., Hwang K. (2016). Photoaging protective effects of BIOGF1K, a compound-K-rich fraction prepared from Panax ginseng. *Journal of Ginseng Research*.

[103] Ishitsuka Y., Maniwa F., Koide C. (2012). Increased halogenated tyrosine levels are useful markers of human skin ageing, reflecting proteins denatured by past skin inflammation. *Clinical and Experimental Dermatology*.

[104] Lee C. S., Bae I.-H., Han J. (2014). Compound K inhibits MMP-1 expression through suppression of c-Src-dependent ERK activation in TNF-*α*-stimulated dermal fibroblast. *Experimental Dermatology*.

[105] Do Y.-K., Kim J.-M., Chang S.-M., Hwang J.-H., Kim W.-S. (2009). Enhancement of polyphenol bio-activities by enzyme reaction. *Journal of Molecular Catalysis B: Enzymatic*.

[106] Miller N. J., Begoña Ruiz-Larrea M. (2002). Flavonoids and other plant phenols in the diet: Their significance as antioxidants. *Journal of Nutritional and Environmental Medicine*.

[107] Choo M.-K., Park E.-K., Han M. J., Kim D.-H. (2003). Antiallergic activity of ginseng and its ginsenosides. *Planta Medica*.

